# SUMO-specific protease 3 is a key regulator for hepatic lipid metabolism in non-alcoholic fatty liver disease

**DOI:** 10.1038/srep37351

**Published:** 2016-11-17

**Authors:** Yuhan Liu, Fudong Yu, Yan Han, Qing Li, Zhujun Cao, Xiaogang Xiang, Shaowen Jiang, Xiaolin Wang, Jie Lu, Rongtao Lai, Hui Wang, Wei Cai, Shisan Bao, Qing Xie

**Affiliations:** 1 Department of Infectious Diseases, Ruijin Hospital, Shanghai Jiaotong University School of Medicine, Shanghai, 200025, China; 2 Department of General Surgery, Shanghai General Hospital, Shanghai Jiaotong University School of Medicine, Shanghai, 200080, China; 3 Discipline of Pathology, School of Medical Sciences and Bosch Institute, University of Sydney, Sydney, NSW 2006, Australia

## Abstract

Non-alcoholic fatty liver disease (NAFLD) is characterized by excessive lipid accumulation in hepatocytes. The role of SENP3 in lipid metabolism, particularly NAFLD, is unclear. Our results showed that hepatic SENP3 was up-regulated in NAFLD patients and an animal model *in vivo* and after loading hepatocytes with free fatty acids (FFA) *in vitro*. Intracellular lipid accumulation was determined in SENP3 silenced or overexpressed hepatocytes with/without FFA *in vitro*. Confirming a role for SENP3, gene silencing was associated *in vitro* with amelioration of lipid accumulation and overexpression with enhancement of lipid accumulation. SENP3 related genes in NAFLD were determined *in vitro* using RNA-Seq. Eleven unique genes closely associated with lipid metabolism were generated using bioinformatics. Three selected genes (*apoe, a2m* and *tnfrsf11b*) were verified *in vitro*, showing *apoe, a2m* and *tnfrsf11b* were regulated by SENP3 with FFA stimulation. Intrahepatic and circulating APOE, A2M and TNFRSF11B were elevated in NAFLD compared with controls. These data demonstrate the important role of SENP3 in lipid metabolism during the development of NAFLD *via* downstream genes, which may be useful information in the development of NAFLD. The precise role of SENP3 in NAFLD will be investigated using liver-specific conditional knockout mice in future studies.

Non-alcoholic fatty liver disease (NAFLD), due to aberrant lipid metabolism, has become an increasingly prevalent health problem worldwide^1^. Anomalous lipid metabolism plays a crucial role in the progression of NAFLD, attacking hepatocytes^2^. Excessive accumulation of triglyceride (TG) in hepatocytes, as a precursor for NAFLD, is due to increased uptake of free fatty acids (FFA) into the liver, up-regulated triglyceride synthesis, impaired lipid β-oxidation, or weakened VLDL secretion^3^. Excessive TG is stored in hepatocytes as lipid droplets. Perturbed metabolic pathways (e.g. mTOR and AKT/PI3K signaling pathway) and abnormal expression of related molecules [e.g. Apo lipoproteins, free acid binding proteins (FABPs), liver X receptors] contribute to the formation of lipid droplets in the onset of steatosis in NAFLD^4^. Hepatic steatosis increases the risk of irreversible liver disease, ultimately leading to liver fibrosis, cirrhosis or hepatocellular carcinoma^5^.

Ubiquitin-like SUMO (small ubiquitin-related modifiers), including SUMO1, SUMO2 and SUMO3, are involved in post-translational modification of target proteins. SUMOs regulate a broad spectrum of cellular processes, e.g. cell signaling, cycle, transcription, or carcinogenesis^6^. SUMOylation is a dynamic process that can be reversed by a family of SUMO-specific proteases (SENPs)^7^. Six SENP members in mammals, identified with different subcellular locations and substrates, play important roles in the control of various cellular events^7^. SENPs, by acting in the protein regulatory event deSUMOylation, play important roles during the progression of many diseases. It has been reported that overexpressed SENP1 promotes mitochondrial biogenesis and function in myotubes^8^. SENP2 inhibits glycolysis, reprograms glucose metabolic strategy in cancer cells^9^ and regulates fatty acid metabolism in skeletal muscle^10^, suggesting an important role of SENP2 in metabolic disorders. SENP5 is reported to maintain mitochondrial morphology and metabolism^11^. As a crucial member of the SENPs, SENP3 is specific to de-conjugate SUMO2 or SUMO3, which share 96% sequence similarity^12^. SENP3 is rapidly increased following several cell stresses e.g. oxidative stress or hyperglycemic stress^13^,^14^. SENP3 is up-regulated in the progression of many diseases, including cancers^13^ and other injuries^15^. Up-regulated SENP3 enhances cell apoptosis/death^16^, proliferation^13^ and cancer metastasis^17^. However, it is unknown whether SENP3 and SENP3-related molecules contribute to the progression of NAFLD *via* manipulation of lipid accumulation in hepatocytes. Thus, the aim of the current study is to explore the role of SENP3 in the development of NAFLD.

## Materials and Methods

### Ethical considerations

All the experiments protocols involving humans and animals were approved by the *Human Ethics Committee of Ruijin Hospital, Shanghai Jiaotong University School of Medicine*. Methods were carried out in accordance with the approved guidelines and regulation. Written informed consent was obtained from all participants. Appropriate care was given to all animals included for experiments.

### Patients

Plasma from NAFLD patients (n = 50) and healthy controls (HCs) (n = 40) [2012 AASLD criteria^5^], identified between Jan 2014 and Oct 2015 in the Department of Infectious Diseases, Ruijin Hospital, Shanghai, China was collected and stored at −80 °C before analysis. Liver biopsies from NAFLD patients (n = 8) and liver transplant donors (n = 3) were also collected in the Department of Infectious Diseases, Ruijin Hospital, Shanghai, China. Paraffin embedded liver tissues were used for immunohistochemistry (IHC).

### Animal studies

Five-week-old male SD rats, each weighing 150–160 g were purchased from the Shanghai Laboratory Animal Company (Shanghai, China). All rats were bred in a specific pathogen-free facility and maintained in a 12-hour light-dark-cycle at room temperature and fed *ad libitum*. The rats were randomly divided into two groups, normal diet (ND) fed with standard chow (n = 5) or high fat diet (HFD) fed with 45% fat chow (n = 5)^18^. The rats were sacrificed on the 60^th^ day. Serum was collected and stored at −80 °C before analysis. Part of the liver tissues were fixed with 10% formalin and embedded in paraffin for routine HE staining and IHC, while other parts were snap frozen and stored in liquid nitrogen until use. Hepatic TG content was determined using the TG assay kit (Applygen Technologies Inc., Beijing, China). In addition, livers from HFD fed 30, 60 and 120 day rats (n = 5, respectively) were collected for Western blotting, and the correlation between hepatic SENP3 and TG was determined.

### Cell culture

Human normal hepatocytes (L02 cell line), obtained from China Cell Culture Center (Shanghai, China), were cultured in Gibco® RPMI 1640 medium supplemented with 10% (v/v) fetal bovine serum (Sigma, MO, USA) under an atmosphere of 5% CO_2_ at 37 °C. Hepatocytes were exposed to free fatty acid (FFA), a mixture of oleic acid and palmitic acid (Sigma, MO, USA), at the final ratio of 2:1, with a total concentration of 0.5 mmol/L for 12 hours to establish steatotic hepatocytes. SENP3-siRNA (siRNA specific for SENP3) and NS-siRNA (non-specific siRNA) oligonucleotides were purchased from Ribobio (Guangzhou, China). The sequences of SENP3-siRNA oligonucleotides were 5′- GGGCUGGAAAGGUUACUUCdTdT-3′ and 3′-dTdTCCCGACCUUUCCAAUGAAG-5′. GFP-SENP3 plasmid and PCDNA3.1 (vehicle plasmid) were kind gifts from the Education Ministry Key Laboratory for Cell Differentiation and Apoptosis, Shanghai Jiaotong University School of Medicine, China^13^,^19^. These hepatocytes were transfected with the siRNA oligonucleotides or plasmids using lipofectamine 2000 (Invitrogen, USA), according to the manufacturer’s instructions. The transfected hepatocytes were harvested with/without 12 hours FFA stimulation for quantification of intracellular lipid accumulation using Oil red O (Sigma, USA) staining^20^, or intracellular TG content using the TG assay kit (Applygen Technologies Inc.).

### RNA-Seq (mRNA sequencing)

Total RNA was extracted from the three hepatocyte treatment groups: FAA treated only, SENP3 silenced with FFA treatment, and SENP3 overexpressed with FFA treatment. Total RNA was isolated using the standard Trizol method and *paired-end library preparations* were performed. RNA-Seq was performed to determine the mRNA expression profile using a large scale, automated variant of the Illumina HiSeq 2500 v4 (Berry Genomics Co., Ltd, Beijing, China). All the clean reads after raw data pre-processing were aligned against the reference genome and transcriptome data downloaded from UCSC (version hg19), using the SOAP2 package^21^. The reads uniquely mapped to a gene to quantified transcript levels were normalized by the RPKM method^22^.

### Analysis of differentially expressed genes (DEGs)

DEGs were identified using the fold change method and adjusted by the generalized fold change algorithm (GFOLD)^23^ between two groups. To identify DEGs, the cut-off value with absolute GFOLD value of 1 was selected. All the scripts used perl and R. All the DEGs were performed against the ExoCarta database^24^ to obtain secreted proteins in plasma or serum. To enrich the lipid related metabolism pathways, all secreted DEGs were evaluated, using the Ingenuity Pathway Analysis (IPA) (Ingenuity® Systems, www.ingenuity.com) against human species. The gene list was generated from the IPA result associated with lipid metabolism and narrowed down with disease functions of steatosis and steatohepatitis.

### Western blotting

The protein concentration was measured following total protein extraction from hepatic tissues or cultured hepatocytes, using RIPA lysis buffer (Beyotime, China). Western blotting was performed, using antibodies against SENP3 (Cell Signaling Technology, MA, USA) and β-actin (Sigma, USA). Protein production was quantitated using Image J.

### Quantitative real-time PCR (qRT-PCR)

qRT-PCR on select genes were performed to verify the bioinformatics derived from RNAseq, as described^25^ using SYBR® Premix Ex Taq^TM^ II (Takara, Dalian, China) and an ABI ViiA7 instrument (Applied Biosystems, USA). Relative expression levels of mRNA were calculated by applying the 2^−ΔΔCt^ method using reference *gapdh* for mRNA and normalized to the control group. Sequences of primers used are outlined in *Supplementary Materials and Methods*.

### Immunohistochemistry (IHC)

Five μm sections of the liver tissues were labelled immunohistochemically and quantitated using Image-Pro Plus 9.0 software (Diagnostic Instruments, Sydney, NSW, Australia) and the data were expressed as image units, as described previously^26^. The primary antibody against SENP3 was purchased from Cell Signaling Technology, and the primary antibodies against APOE, A2M and TNFRSF11B (also named Osteoprotegerin, OPG) were purchased from Abcam, Cambridge, MA, USA.

### ELISA

Circulating levels of A2M, APOE and TNFRSF11B were measured according to the instructions from the manufacturers (Abcam, MA, USA).

### Statistical analysis

Values were presented as mean ± SEM. All statistical analyses were carried out using GraphPad Prism 5 and Sigmaplot 12.5 for Windows. Statistically significance was regarded as *p < 0.05, **p < 0.01 and ***p < 0.001.

## Results

### SENP3 was significantly up-regulated in the livers from NAFLD patients and HFD fed rats

**Figure 1:**
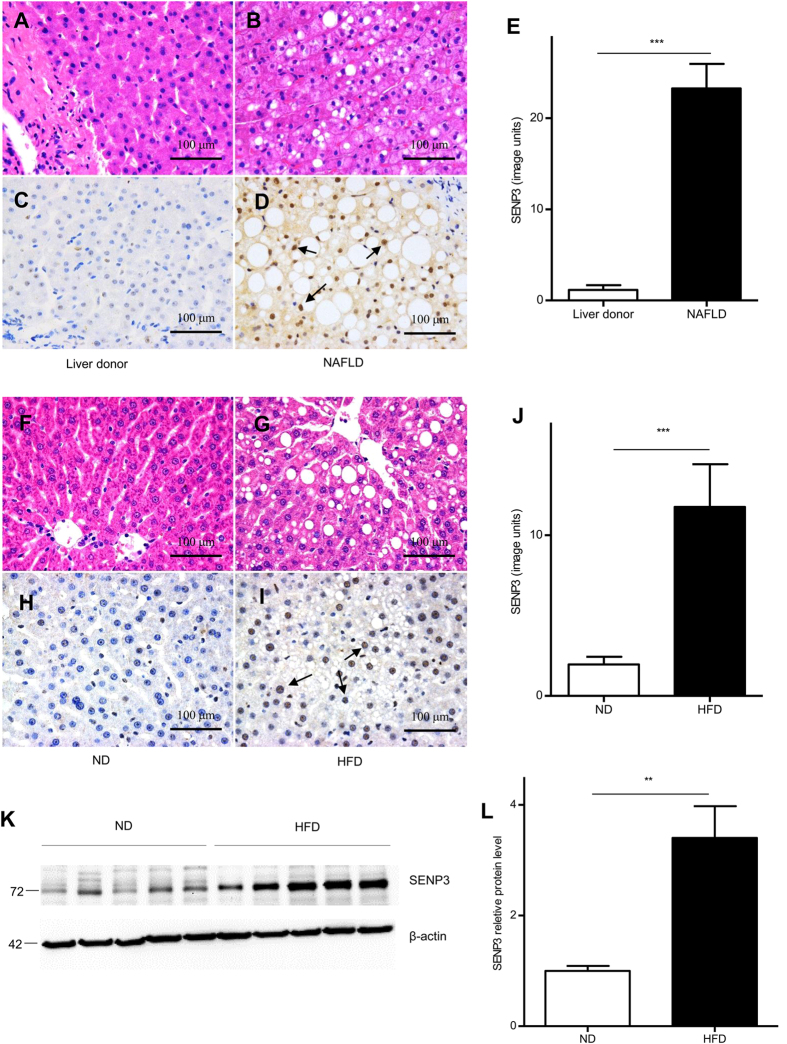
Intrahepatic SENP3 production was determined in NAFLD and controls. Histopathology of the liver tissues from healthy liver donors (n = 3) (**A**) and NAFLD (n = 8) (**B**) were H&E stained. Intrahepatic SENP3 production from healthy liver donors (**C**) and NAFLD (**D**) were labelled immunohistochemically, with the black arrows indicated the positive labelling. Quantification of immune-staining was presented (**E**, *p* < 0.001). The corresponding H&E of the liver from ND- (n = 5) (**F**) and HFD-fed (n = 5) (**G**) rats and immunohistochemistry of SENP3 in the liver from ND- (**H**) and HFD-fed (**I**) rats and their corresponding quantification were also presented (**J**, *p* < 0.001). The hepatic protein production of SENP3 from HFD-fed and ND-fed rats was confirmed by Western blotting (**K**) and the quantification was presented (L, *p* < 0.01).

To investigate if SENP3 was modified in NAFLD, the production of SENP3 was examined in livers from liver transplant donors and NAFLD patients. Histopathology of the liver tissues from liver donors (Fig. 1A) and NAFLD (Fig. 1B), and immunohistochemistry for SENP3 in livers from liver donors (Fig. 1C) and NAFLD (Fig. 1D) were stained. SENP3 was mainly localized around the nuclei of hepatocytes, consistent with high levels of fatty deposition. The relative mean density of SENP3 was ~20 fold higher in the livers from NAFLD than that in livers from donors (Fig. 1E, *p* < 0.001), suggesting the importance of SENP3 during the development of NAFLD. Due to lack of sufficient human liver tissue for further confirmation of SENP3 for Western blotting, an animal model of NAFLD was established by feeding SD rats a HFD for 60 days. Serum alanine aminotransferase (ALT), but not aspartate aminotransferase (AST), was significantly higher in HFD-fed than ND-fed rats (Supplementary Figure 1A, *p* < 0.05, and Supplementary Figure 1B). Both serum TG (Supplementary Figure 1C, *p* < 0.05) and intrahepatic TG (Supplementary Figure 1D, *p* < 0.01) were significantly higher in HFD-fed rats than in ND-fed rats. Representative photomicrographs of the liver were shown following HE staining of ND-fed (Fig. 1F) and HFD-fed (Fig. 1G) rats and IHC of SENP3 in livers from ND-fed (Fig. 1H) and HFD-fed (Fig. 1I) rats. The relative mean density of intrahepatic SENP3 was ~10 fold higher in HFD-fed rats compared with ND-fed rats (Fig. 1J, *p* < 0.01). Significant up-regulation of SENP3 in livers from HFD-fed rats compared with that from ND-fed rats (Fig. 1K) (Fig. 1L, *p* < 0.01), was confirmed using Western blotting.

Hepatic SENP3 in rats fed a HFD for 0, 30, 60 and 120 days was determined by Western blotting (Supplementary Figure 2A). SENP3 relative protein level was gradually up-regulated following HFD over 120 days, but appeared to plateau at day 60 (Supplementary Figure 2B). Furthermore, there was a significant correlation between hepatic SENP3 and TG (rho = 0.77, p < 0.001) (Supplementary Figure 2C). However, there was no significant correlation between hepatic SENP3 and severity of steatosis [(mild, 5–33%, n = 3) and (moderate, 33–66%, n = 5)] NAFLD patients (data not shown). The data *in vivo* above suggests a close relationship between SENP3 and NAFLD, which invites speculation that SENP3 is correlated with the development of NAFLD.

### SENP3 contributed to the severity of hepatic steatosis *in vitro*

**Figure 2:**
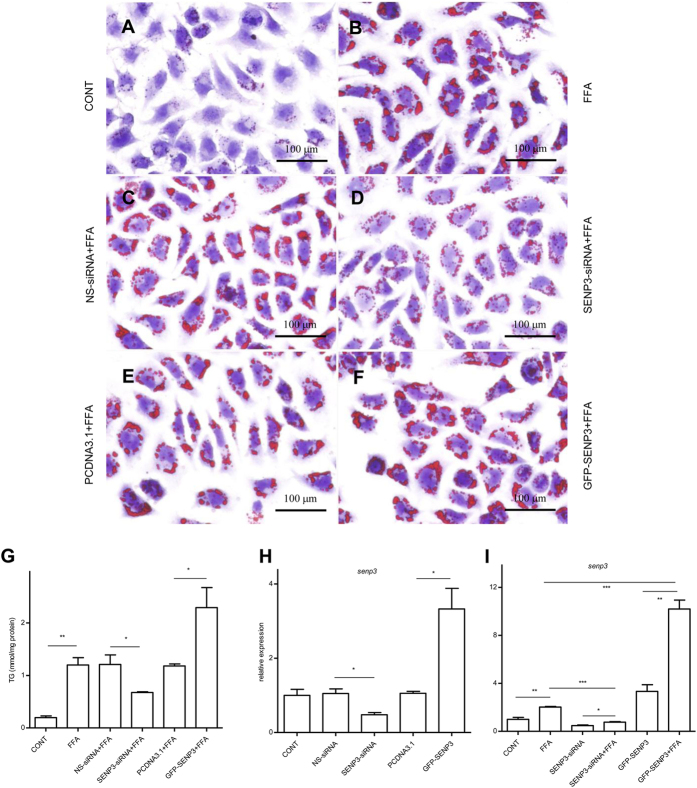
SENP3 regulated lipid accumulation in hepatocytes. Intrahepatic lipid was labelled with Oil red o in hepatocytes treated without FFA (**A**) and with FAA (**B**). Compared with NS-siRNA (**C**), FFA induced lipid accumulation was attenuated in hepatocyte transfected with SENP3-siRNA (**D**). Compared with PCDNA3.1 (**E**), FFA induced lipid content was further enhanced with GFP-SENP3 (**F**). Intrahepatic TG was quantified from different treatments (**G**). Intrahepatic SENP3 mRNA from the different treatments was quantified, using qRT-PCR (**H**,**I**).

A model of hepatic steatosis was established in cultured hepatocytes to further explore the role of SENP3 in NAFLD *in vitro*. Compared with non-treated hepatocytes (Fig. 2A), intracellular lipid accumulation was substantially up-regulated in FFA-treated hepatocytes (Fig. 2B), using Oil Red O staining. Consistent with the *in vivo* findings above, significantly up-regulated SENP3 protein was detected in FFA-treated hepatocytes compared with non-treated hepatocytes *in vitro*, using Western blotting (Supplementary Figure 3A, B, *p* < 0.01). To verify if SENP3 contributes to the development of NAFLD, SENP3 was either silenced with SENP3-siRNA (Supplementary Figure 3C) or overexpressed with a GFP-SENP3 plasmid (Supplementary Figure 3D) in hepatocytes *in vitro*. Subsequently, these genetically manipulated cells were further treated with or without FFA for 12 hours. Compared with NS-siRNA (Fig. 2C), up-regulated lipid accumulation with FFA treatment was markedly ameliorated with SENP3-siRNA transfection (SENP3 inhibition) (Fig. 2D); whereas, compared with PCDNA3.1 (Fig. 2E), lipid accumulation was substantially increased with GFP-SENP3 transfection (SENP3 overexpression) (Fig. 2F). Consistent with our hypothesis, SENP3 silencing or overexpression resulted in a significant reduction (44%, *p* < 0.05) or up-regulation (~2 fold, *p* < 0.05) of cellular TG compared to NS-siRNA or PCDNA3.1 vehicle plasmid transfected group following FFA exposure (Fig. 2G). Collectively, these data suggest that SENP3 plays a critical role in steatosis.

qRT-PCR was applied to verify the expression of SENP3 in hepatocytes with different treatments. SENP3 was significantly up-regulated in GFP-SENP3 transfected hepatocytes (~3 fold, *p* < 0.05), whereas it was significantly down-regulated with SENP3-siRNA transfection (>50%, *p* < 0.05) (Fig. 2H). SENP3 mRNA level was up-regulated with FFA stimulation in mock-treated hepatocytes (*p* < 0.01), SENP3-siRNA transfected hepatocytes (*p* < 0.05) or GFP-SENP3 transfected hepatocytes (*p* < 0.01), compared with non-FAA-stimulated cells (Fig. 2I). As expected, we observed a 66% down-regulation in SENP3 in SENP3 silenced hepatocytes with FAA stimulation, compared with the non-treated hepatocytes with FFA stimulation (Fig. 2I, *p* < 0.01). On the other hand, 5 fold up-regulated SENP3 was detected in SENP3 overexpressed hepatocytes with FAA, compared with the non-treated hepatocytes with FFA stimulation (Fig. 2I, *p* < 0.01). Overall, these data provide further evidence that SENP3 plays an important role in steatosis.

### Differentially expressed genes (DEGs) in NAFLD

**Figure 3:**
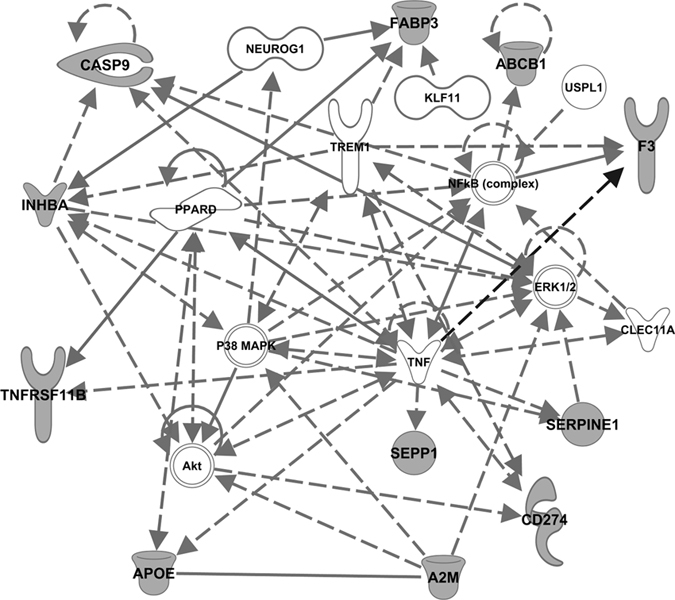
The sub-network of 11 secreted lipid metabolic disorder related genes were identified with NAFLD functional selection. The bold nodes represented 11 recognized secreted genes.

To explore the role of SENP3 during the development of NAFLD, the expression of SENP3 and SENP3-related genes were determined during the development of NAFLD *in vitro*, using the RNA-Seq method. DEGs were detected using the GFOLD algorithm adjusting among the SENP3 silenced, overexpressed and the mock-gene treated groups following FFA stimulation. There were 140 DEGs identified in the SENP3 overexpressed hepatocytes following FFA treatment, compared to the mock-gene treated group, and 426 DEGs in SENP3 silenced hepatocytes following FFA treatment, compared to the mock-gene treated group. After combining these DEGs from the groups with overexpression and silencing of SENP3, 532 unique DEGs were generated. To determine which of these DEGs were of clinical value in NAFLD patients, potential secreted proteins among these DEGs were selected by searching against the ExoCarta diagnosis and prognosis database to find those likely to be secreted into plasma or serum^24^. It was identified that 91 out of 532 DEGs were potential secreted proteins (Supplementary Table 1). To enrich these DEGs functions, these 91 secreted genes were further submitted to the IPA pathway package against human species to accumulate the potential sub-network(s) or pathway(s) related to lipid metabolism (Supplementary Figure 4). Using NAFLD functional selection, it was further recognized that 11 out of 91 unique secreted genes were related to liver steatosis and steatohepatitis (Table 1). A sub-network enriched for lipid metabolic disorder was constructed based on these 11 recognized genes (Fig. 3). These secreted proteins might be potentially promising diagnostic biomarkers of NAFLD. The GFOLD value from the DEGs overexpression groups revealed the top 3 SENP3-related genes (*apoe, a2m* and *tnfrsf11b*), which were then utilized to confirm the clinical significance of SENP3 activity (Fig. 3).

### A*poe, a2m* and *tnfrsf11b* were regulated by SENP3 following FFA treatment in hepatocytes

**Figure 4:**
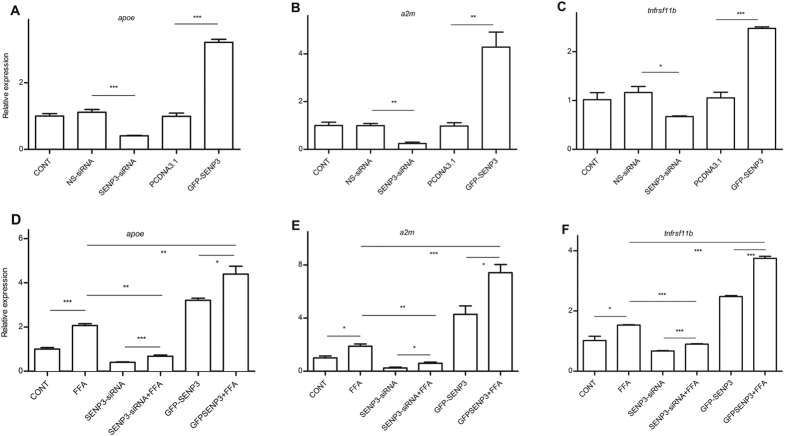
Top three SENP3 related genes (*apoe, a2m*, and *tnfrsf11b*) were quantified *in vitro* using qRT-PCR. mRNA of *apoe* (**A**), *a2m* (**B**), and *tnfrsf11b* (**C**) in the hepatocytes was silenced with SENP3-siRNA transfection, but increased with GFP-SENP3 transfection. To determine the effects of SENP3 in lipid regulation, *apoe* (**D**), *a2m* (**E**) and *tnfrsf11b* (**F**) mRNA levels were further quantified in control, SENP3-siRNA transfected and GFP-SENP3 transfected hepatocytes with/without FFA stimulation.

Expression of *apoe, a2m* and *tnfrsf11b* was confirmed using qRT-PCR in hepatocytes. The *apoe* mRNA level was significantly inhibited (~60%, *p* < 0.001) in SENP3-siRNA transfected (SENP3 knocked-down) hepatocytes compared with NS-siRNA transfected hepatocytes (Fig. 4A). On the other hand, *apoe* was significantly up-regulated (>3 fold, *p* < 0.001) in GFP-SENP3 transfected (SENP3 overexpressed) hepatocytes compared with PCDNA3.1 transfected hepatocytes (Fig. 4A). Similarly, mRNA levels of *a2m* (Fig. 4B) and tnfrsf11b (Fig. 4C) were significantly inhibited with SENP3 silencing, or increased with SENP3 overexpression, consistent with the regulatory role of SENP3. There was also a significant up-regulation of *apoe* following FFA stimulation in mock-treated hepatocytes (*p* < 0.001), SENP3-silenced hepatocytes (*p* < 0.01) or SENP3-overexpressed hepatocytes (Fig. 4D, *p* < 0.05), consistent with data obtained from SENP3 expression above. Up-regulated *apoe* was detected in SENP3-overexpressed hepatocytes plus FAA stimulation, compared with mock-treated hepatocytes plus FFA stimulation (Fig. 4D, >2 fold, *p* < 0.01). As expected, ~70% down-regulation of *apoe* was detected in SENP3-silenced hepatocytes following FAA stimulation, compared with mock-treated hepatocytes plus FFA stimulation (Fig. 4D, *p* < 0.001). Interestingly, the constitutive expression of *a2m* (Fig. 4E) and *tnfrsf11b* (Fig. 4F) in hepatocytes was also significantly up-regulated with FFA stimulation. Moreover, *a2m* (Fig. 4E) and *tnfrsf11b* (Fig. 4F) were down-regulated in SENP3-silenced hepatocytes plus FFA, compared with mock-treated hepatocytes plus FFA, and up-regulated in SENP3-overexpressed hepatocytes plus FFA, compared with mock-treated hepatocytes plus FFA. The mRNA expression of *a2m* or *tnfrsf11b* modified by either SENP3 silencing or over expression with FFA was in line with the findings in *apoe* gene, but at different magnitudes. Taken together, mRNA levels of *apoe, a2m* and *tnfrsf11b*, regulated by SENP3, were up-regulated following FFA stimulation *in vitro*. Thus, these data are consistent with SENP3, contributing to hepatic steatosis *via* the 3 selected lipid metabolism-related genes.

### Up-regulated intrahepatic and circulating APOE, A2M and TNFRSF11B in NAFLD patients

**Figure 5:**
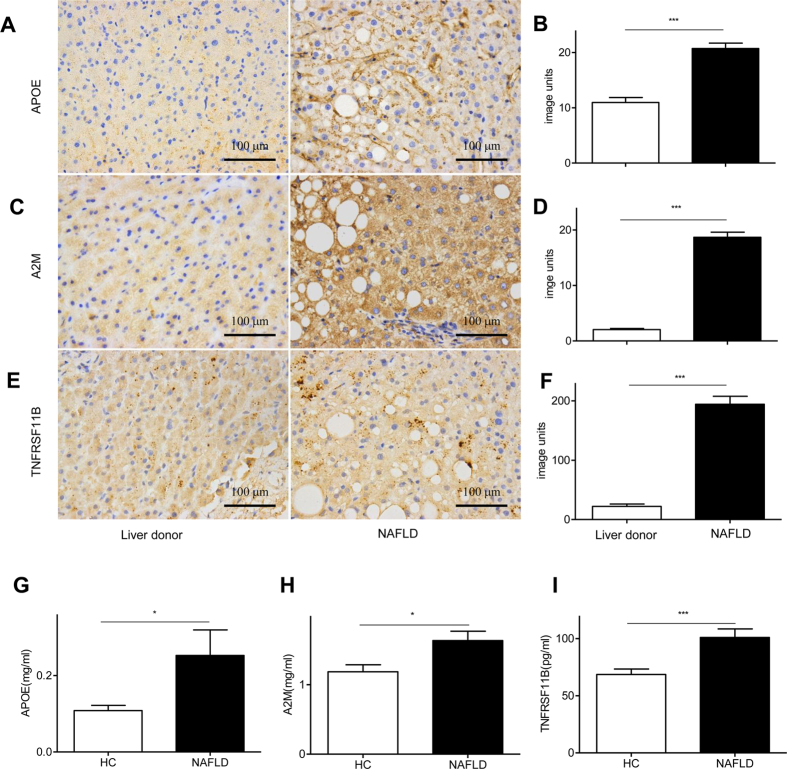
Intrahepatic and circulating APOE, A2M, and TNFRSF11B were determined in NAFLD patients and controls. APOE (**A**), A2M (**C**), and TNFRSF11B (**E**) in the liver tissues from healthy liver donors (left panels, n = 3) and NAFLD (right panels, n = 8) were labelled immunohistochemically. The quantification of APOE (**B**, *p* < 0.001), A2M (**D**, *p* < 0.001), and TNFRSF11B (**F**, *p* < 0.001) were also presented. Circulating levels of APOE (**G**, *p* < 0.05), A2M (**H**, *p* < 0.05), and TNFRSF11B (**I**, *p* < 0.001) in NAFLD patients (n = 50) and HCs (n = 40) were verified by ELISA.

Following the data obtained in hepatocytes at mRNA level, the corresponding protein levels of *apoe, a2m* and *tnfrsf11b* were determined in the liver tissue from NAFLD patients and healthy liver transplant donors, as well as in the plasma from NAFLD patients and HCs. Constitutive protein production of APOE was observed in livers from healthy liver transplant donors (Fig. 5A, left) and NAFLD (Fig. 5A, right) patients. In NAFLD patients, APOE was mainly located in the cell membrane of hepatocytes and the adjacent extra-cellular space, particularly in regions containing many fat vacuoles. APOE was significantly increased (Fig. 5B, *p* < 0.001) in the hepatocytes from NAFLD patients, compared to those from the donors. A2M (Fig. 5C) and TNFRSF11B (Fig. 5E) in the liver from the donor (left panels) and NAFLD patients (right panels) were also detected immunohistochemically, with increased staining in NAFLD patients mainly located in the cytoplasm. A2M (Fig. 5D, *p* < 0.001) and TNFRSF11B (Fig. 5F, *p* < 0.001) were significantly up-regulated, compared with liver transplant donors. Thus, the expression of all three proteins was increased in liver tissue samples from NAFLD patients.

Liver biopsy is an invasive procedure during clinical practice, thus an alternative diagnostic approach is desirable to measure circulating APOE, A2M and TNFRSF11B. Hence, we used ELISA to measure circulating levels of these proteins. The circulating level of APOE was 2.5 fold higher in NAFLD patients than from HCs (Fig. 5G, *p* < 0.05). Similarly, circulating A2M (Fig. 5H, 1.38 fold, *p* < 0.05) and TNFRSF11B (Fig. 5I, 1.47 fold, *p* < 0.001) were significantly higher in NAFLD patients than HCs. These plasma data are consistent with the immunohistochemical findings in the liver tissues.

## Discussion

In the current study we observed that SENP3 was increased in the liver from NAFLD patients and HFD-fed rats *in vivo*, as well as FFA-treated hepatocytes *in vitro*. Lipid accumulation in FFA-stimulated hepatocytes was inhibited with SENP3 silencing, but was enhanced with SENP3 overexpression. SENP3 related genes in NAFLD were generated using RNA-Seq from the hepatocytes *in vitro*. The top three genes (*apoe, a2m* and *tnfrsf11b*) were found to be regulated by SENP3 in hepatic steatosis *in vitro*. Furthermore, protein levels of APOE, A2M and TNFRSF11B were significantly up-regulated in the liver and plasma of NAFLD patients compared with the controls. Such data demonstrate a clear role for SENP3 in lipid metabolism during the development of NAFLD.

Mammals possess six SENP isoforms that are capable of reversing SUMO-1, -2 and -3 post-translational modifications. These isoforms play different roles in the control of various cellular events^27^. SENP2 regulates adipogenesis *via* de-SUMOylation of C/EBPβ, which in turn promotes C/EBPα and PPARγ^28^, and controls glucose metabolism *via* p-AKT^9^. Overexpression of SENP2 promotes fatty acid metabolism in skeletal muscle *via* fatty acid oxidation, ultimately alleviating obesity-linked metabolic disorders^10^. On the other hand, SENP2 silence attenuates adipogenesis in preadipocytes^28^. In addition, down-regulation of SENP2 increases SUMOylation of p53 and ERK5, leading to atherosclerotic plaque formation^29^. In the current study, hepatic production of SENP3 was significantly higher in NAFLD patients and HFD-fed rats than healthy liver transplant donors or ND-fed rats, as well as, in FFA-treated hepatocytes than non-treated cells.

A close correlation between hepatic TG and SENP3 in HFD fed rats suggests a possible role of SENP3 in lipid metabolism in NAFLD. However, the lack of a significant correlation between the severity of steatosis and hepatic SENP3 from NAFLD patients might be due to relatively small numbers of patients in this study and/or patient polymorphisms. There are both clinical and ethical challenges to performing liver biopsies in NAFLD patients. The large number of liver biopsies required for our future clarification of the correlation between the severity of steatosis and hepatic SENP3 are currently being collected.

The observation of the correlation between SENP3 and NAFLD in patients and the animal model *in vivo* was further clarified in hepatocytes *in vitro*. It was confirmed that increased lipid accumulation in FFA-treated hepatocytes was attenuated or enhanced with SENP3 silencing or overexpression, respectively, arguing for the regulation of hepatic steatosis by SENP3. The corresponding mRNA levels of SENP3 were also confirmed. Thus, the data delineated that SENP3 activity contributes to the severity of steatosis, suggesting a potential novel therapeutic target for the management of hepatic steatosis. We observed that SENP3 is up-regulated in livers from NAFLD patients and HFD fed rats *in vivo*, and after loading hepatocytes with free fatty acids (FFA) *in vitro*. We also observed that SENP3 overexpression resulted in a significant up-regulation of cellular TG compared with control group following FFA exposure. Thus, we believe that SENP3 likely acts in both paracrine and autocrine fashions during steatosis. However, the precise underlying mechanism of SENP3 in steatosis will be determined in future experiments, using SENP3 liver-specific knockout mice.

Subsequently, the differential expression of 11 SENP3-related genes were identified in the NAFLD disease state, following functional selection from 91 secreted genes out of 532 DEGs. Three out of these 11 genes (*apoe, a2m* and *tnfrsf11b*) were subsequently selected to validate their significance in NAFLD. Expression of *apoe, a2m* and *tnfrsf11b* genes were elevated in steatotic hepatocytes and were regulated by SENP3 at the mRNA level. APOE, a ligand for lipoprotein receptors, regulates lipid metabolism *via* participating in lipid transportation and promoting lipid accumulation^30^. The critical role of APOE in metabolic syndrome has been well documented in APOE gene knock-out mice^31^. Interestingly, APOE, regulated by SENP3, was elevated in steatotic hepatocytes, as well as, in the liver and plasma from the NAFLD patients. There is a close correlation between elevated APOE and hyperlipidemia in metabolic syndrome patients^32^. We speculate that the elevated APOE in NAFLD may be regulated by SENP3 indirectly. The up-regulation of APOE may be a response by the body to metabolize the elevated lipid levels in the liver of the NAFLD patients we examined. The APOE response in NAFLD may be functionally insufficient or inefficient, which is being currently investigated.

A2M can promote cell proliferation by increasing glucose uptake, lactate secretion and lipogenesis, as an insulin-like response^33^. Abnormal up-regulation of A2M is associated with intracellular lipid accumulation in a new model of NAFLD^34^. Such findings are consistent with the data obtained in our current study, i.e. the observation of significantly elevated circulating and intrahepatic A2M in NAFLD patients, as well as, in steatotic hepatocytes *in vitro*. Our data imply that A2M, regulated by SENP3, plays an important role in the progression of NAFLD.

TNFRSF11B (Osteoprotegerin, OPG) is a secreted protein involved in bone turnover, due to its role as a decoy receptor for the receptor activator of nuclear factor-kB ligand in the osteoclasts^35^. It has been reported that there is a linkage between OPG and NAFLD, particularly in osteoporosis patients^36^, suggesting a possible role of OPG in the development of NAFLD. Our current study showed that OPG was substantially up-regulated at the mRNA level in steatotic hepatocytes *in vitro* and at the protein level in NAFLD patients. Our data suggest that OPG contributes to lipid metabolism in NAFLD, which is in line previous findings^36^.

In addition to *apoe, a2m* and *tnfrsf11b*, substantial alterations in the expression of several other genes were identified during our sub-network analysis (Fig. 3). Relevant examples are as follows. ATP-binding cassette transporter 1 (*abcb1*), which participates in drug metabolism, is a monitor for the efficacy and safety of treatment of NAFLD^37^,^38^. *Caspase-9 (casp9*), is a potential indicator for hepatocyte apoptosis during the development of NAFLD *via* the mitochondrial pathway^39^. FABP3 is a regulator of lipid metabolism and participates in the transport of lipids^40^, and has been used as a biomarker for metabolic syndrome related atherosclerosis in patients with glucose impairment^41^. *Serpine 1* (plasminogen activator inhibitor-1), which correlates to body-mass index, plasma triglyceride and insulin levels, has a significant clinical value in metabolic disturbance related diseases, including atherosclerosis, type 2 diabetes, obesity and liver steatosis^42^,^43^. Our findings suggest that SENP3 plays an important role in homeostasis of lipid metabolism, and dysregulation of SENP3 contributes to consequent NAFLD *via* gene regulation e.g. *apoe, a2m* and *tnfrsf11b*.

There are limitations in the current study. Firstly, the direct linkage between SENP3 and NAFLD has not been fully established in a SENP3 knockout animal model, due to embryonic lethality. However, such a lethal consequence further supports the critical pathophysiological role of SENP3 in homeostasis. Secondly, a larger patient cohort, including population diversity, is desirable for the validation of the 11 selected secreted genes with potential diagnostic values in NAFLD. Thirdly, the precise underlying mechanism of SENP3 regulation during the development of NAFLD is currently being investigated.

The focus of the current study was to investigate whether SENP3 contributes to lipid metabolism in NAFLD, based on our unexpected preliminary observation that SENP3 is up-regulated in fatty liver. Subsequently, follow up *in vivo* and *in vitro* experiments demonstrated a link between SENP3 and NAFLD. There are a number of targets of SENP3 e.g. MEF2D, EP300, RbBP5, NPM1, etc, involved in transcriptional activation, potentiating cell survival and redox regulation^44^. Three molecules (*apoe, a2m* and *tnfrsf11b*) chosen in the current study were based on the data from bioinformatics, which offered the highest probability of a linkage between SENP3 and these three molecules. We also observed differential regulation of *apoe, a2m* and *tnfrsf11b* following manipulation of SENP3. We acknowledge that the underlying mechanism is still unclear. Nevertheless, our current observations suggest that SENP3 regulation of lipid metabolism in fatty liver may possibly be via *apoe, a2m* and *tnfrsf11b*. In addition, it has been reported that SENP3 serves as a redox sensor to enhance HIF1A transcriptional activity by de-SUMOylating EP300 45,46. We will use liver-specific conditional SENP3 knockout mice to investigate the precise role of SENP3 in future experiments.

In conclusion, we observed for the first time that SENP3 was up-regulated in NAFLD patients and an animal model *in vivo*, as well as steatotic hepatocytes *in vitro*. Moreover, SENP3-mediated steatosis occurs possibly *via* regulating downstream genes involved in abnormal lipid metabolism. Such data might shed light on the pathogenesis of steatosis, which may be used as a potential therapeutic target for prevention and/or treatment of NAFLD. The precise role of SENP3 in the development of NAFLD will continue to be studied.

## Additional Information

**How to cite this article**: Liu, Y. *et al*. SUMO-specific protease 3 is a key regulator for hepatic lipid metabolism in non-alcoholic fatty liver disease. *Sci. Rep.* **6**, 37351; doi: 10.1038/srep37351 (2016).

**Publisher’s note:** Springer Nature remains neutral with regard to jurisdictional claims in published maps and institutional affiliations.

## Supplementary Material

Supplementary Information

## Figures and Tables

**Table 1:** The gene expression and potential clinical application of 11 selected secreted genes.

Gene Symbol	OE_Gfold	OE_Log2fdc	OE_group^1^	KD_Gfold	KD_Log2fdc	KD_group^2^	Location	biomarker	Type
A2M	1.52154	1.64718	(12.9488)/(4.10322)	−4.26924	−4.79629	(0.151817)/(4.10322)	extracellular space		transporter
TNFRSF11B	1.54377	1.80015	(6.52981)/(1.85577)	−1.39613	−1.86165	(0.533395)/(1.85577)	plasma membrane	diagnosis	transmembrane receptor
APOE	1.14086	1.55592	(4.24542)/(1.42182)	−0.92172	−1.61638	(0.478918)/(1.42182)	extracellular space	diagnosis, progression	transporter
SEPP1	0.528556	0.768941	(4.82016)/(2.80529)	−1.7658	−2.19251	(0.641311)/(2.80529)	extracellular space		other
CASP9	0.452835	0.545562	(27.7131)/(18.8567)	−1.6161	−1.76155	(5.86863)/(18.8567)	cytoplasm	diagnosis	peptidase
FABP3	0.305249	0.37913	(88.3177)/(67.4482)	−1.61108	−1.72339	(21.5628)/(67.4482)	cytoplasm	diagnosis	transporter
F3	0.06848	0.142588	(36.7201)/(33.0407)	1.56437	1.62653	(107.78)/(33.0407)	plasma membrane		transmembrane receptor
SERPINE1	−0.21564	−0.3676	(5.48462)/(7.03013)	1.38929	1.50678	(21.1157)/(7.03013)	extracellular space	diagnosis, progression, prognosis	other
CD274	0	0.399799	(0.178413)/(0.132517)	1.91475	2.59352	(0.876092)/(0.132517)	plasma membrane		enzyme
ABCB1	0	−0.82259	(0.0369124)/(0.0675486)	−1.16348	−3.15243	(0.00427968)/(0.0675486)	plasma membrane	prognosis	transporter
INHBA	0	0.144985	(0.0889291)/(0.0781141)	1.8329	3.13707	(0.807319)/(0.0781141)	extracellular space	diagnosis	growth factor

Group information: OE_group^1^ comprises SENP3-overexpressed hepatocytes following FFA treatment and mocked-gene-treated hepatocytes following FFA treatment. KD_group^2^ comprises SENP3-silenced hepatocytes following FFA treatment and mocked-gene-treated hepatocytes following FFA treatment. Gfold value with 0 means no statistical significance.
