# Supplementary material for: SUMO-specific protease 3 is a key regulator for hepatic lipid metabolism in non-alcoholic fatty liver disease
Source: Sci Rep. 2016 Nov 17;6:37351. doi: 10.1038/srep37351 (PMC5112590; doi:10.1038/srep37351)
Supplement: Supplementary Information [file srep37351-s1.docx]

**SUMO-specific protease 3 is a key regulator for hepatic lipid metabolism in non-alcoholic fatty liver disease**

Yuhan Liu, Fudong Yu, Yan Han, Qing Li, Zhujun Cao, Xiaogang Xiang, Shaowen Jiang, Xiaolin Wang, Jie Lu, Rongtao Lai, Hui Wang, Wei Cai, Shisan Bao, Qing Xie

**Supplementary Materials and Methods**

Sequences of primers for qRT-PCR were:

*senp3*: 5’-GGATGCTGCTCTACTCAAAAAGC- 3’,

5’-GGGAGTCAAAACGACAACAGG-3’;

*apoe*: 5’-GTTGCTGGTCACATTCCTGG-3’,

5’-GCAGGTAATCCCAAAAGCGAC-3’;

*a2m*: 5’-CGGAGAATGACGTACTCCACT-3’,

5’-TGGGTTGGTCCTTTCACTTGG-3’;

*fabp3*: 5’-CATGACCAAGCCTACCACAAT-3’,

5’-CCCCAACTTAAAGCTGATCTCTG-3’;

*gapdh*: 5’-GAAGGTCGGAGTCAACGGAT-3’,

5’-CCTGGAAGATGGTGATGGG-3’.

**Supplementary Figures**

**

**

**Supplementary Fig. 1** **Measurements of serum ALT, AST, TG and hepatic TG levels in HFD- and ND- fed rats.** Serum ALT is significantly higher in HFD-fed rats than that in ND-fed rats (A). Serum AST is higher in HFD-fed rats than in ND-fed rats, without significant difference (B). Both serum (C) and intrahepatic TG (D) were significantly higher in HFD-fed rats than in ND-fed rats. ^*^*p*<0.5, ^**^*p*<0.01.

**
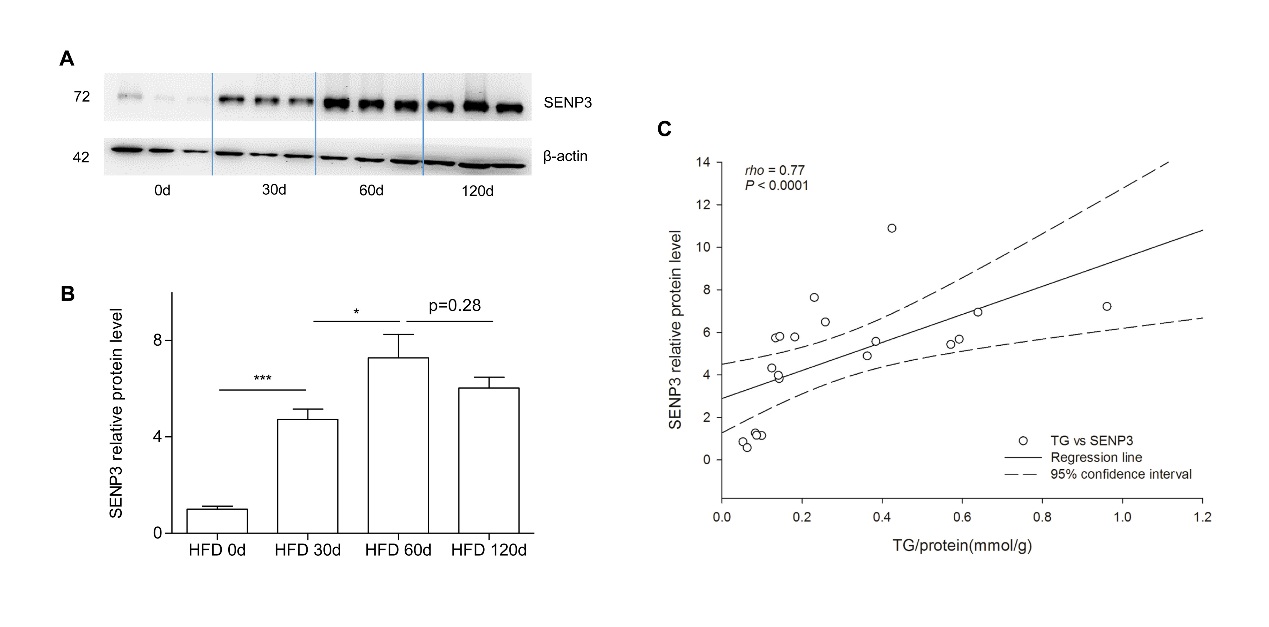
**

**Supplementary Fig. 2 The correlation between SENP3 expression and the severity of steatosis in rats with HFD for different feeding period.** SENP3 production was determined in livers from rats fed with HFD for 0, 30, 60 and 120 days (n=5 for each group). Representative pictures by western blotting was shown (A). Protein production levels were detected by Image J (B). The correlation between SENP3 and TG in the liver was detected by (C).


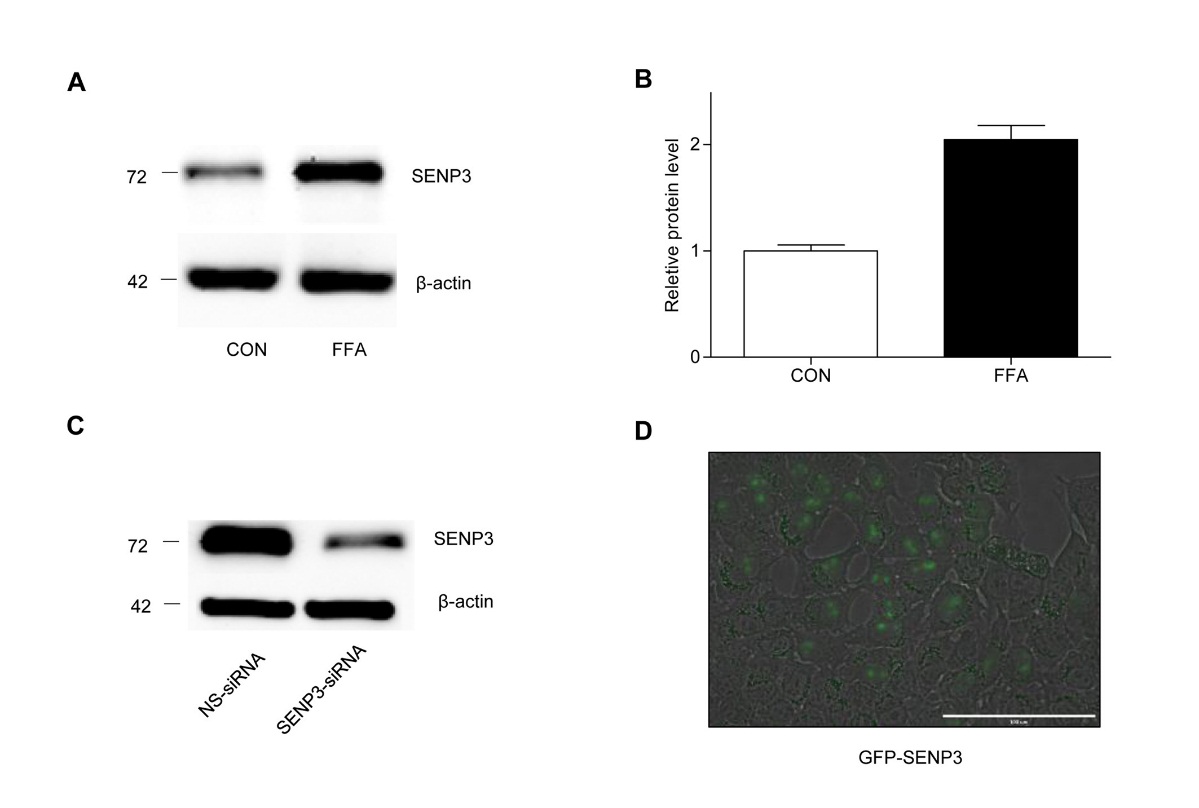


**Supplementary Fig. 3 SENP3 production was determined in hepatocytes.** Western blot showed that SENP3 was upregulated with FFA treatment in hepatocutes (A). Protein production levels were significantly higher (>2 fold, ^**^*p*<0.01) in FFA stimulated hepatocytes than mock-treated hepatocytes (B). SENP3-siRNA transfection significantly reduced SENP3 expression in hepatocytes determined by western blotting (C). Image of hepatocytes transfected with GFP-SENP3 plasmid with fluorescence microscope (D).

**
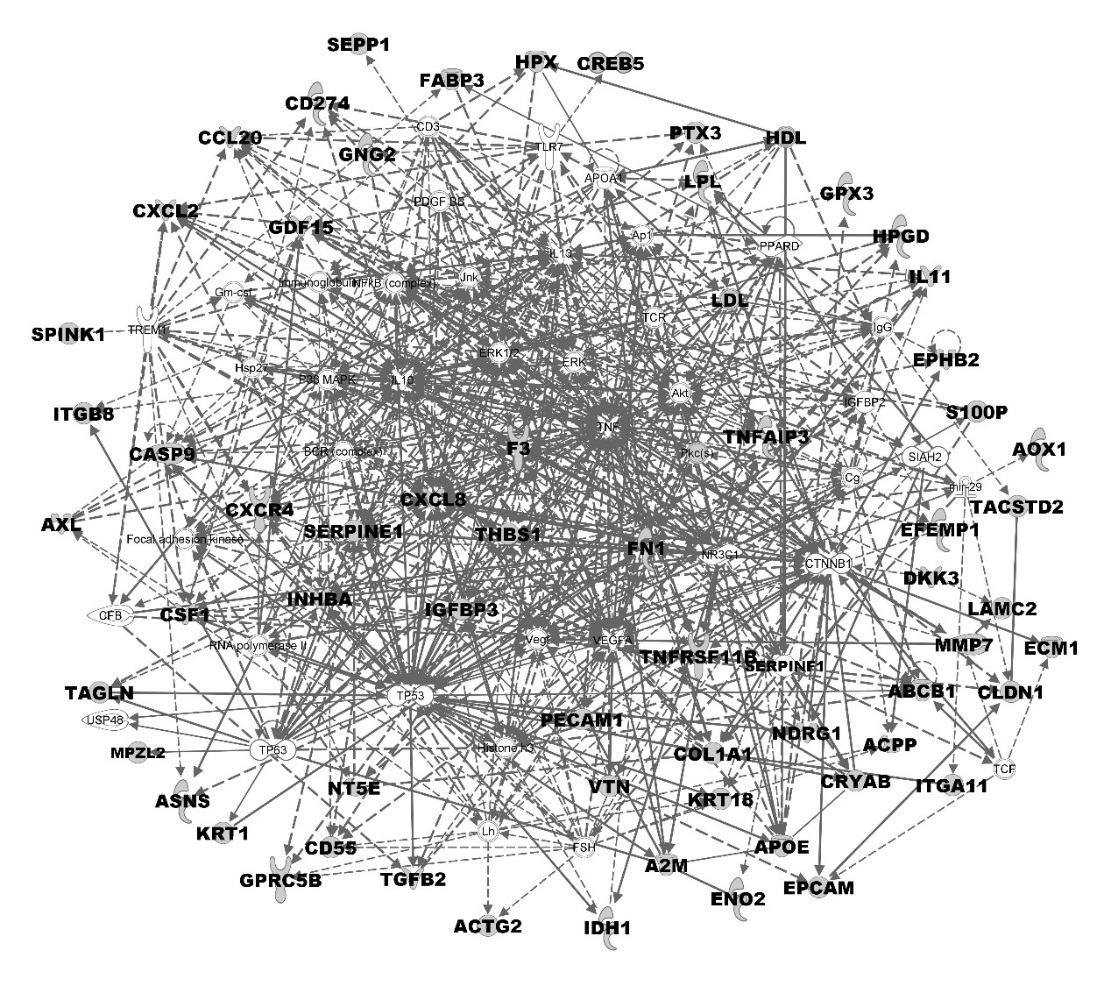
Supplementary Fig. 4** **The sub-network of the 91 secreted genes were submitted to IPA pathway package.**

**Supplementary Table 1 The expression, location and type of 91 secreted genes out of 532 DEGs.**

| GeneSymbol | OE_Gfold | OE_Log2fdc | OE_group^1^ | KD_Gfold | KD_Log2fdc | KD_group^2^ | Location | Type |
| --- | --- | --- | --- | --- | --- | --- | --- | --- |
| A2M | 1.52154 | 1.64718 | (12.9488)/(4.10322) | -4.26924 | -4.79629 | (0.151817)/(4.10322) | Extracellular Space | transporter |
| ABCB1 | 0 | -0.822594 | (0.0369124)/(0.0675486) | -1.16348 | -3.15243 | (0.00427968)/(0.0675486) | Plasma Membrane | transporter |
| ACPP | 0.0932538 | 0.703475 | (0.405282)/(0.244746) | 1.22841 | 1.76511 | (0.893379)/(0.244746) | Extracellular Space | phosphatase |
| ACTG2 | 1.46122 | 1.84341 | (5.17339)/(1.42007) | 0.261764 | 0.695564 | (2.44137)/(1.42007) | Cytoplasm | other |
| AKR1B10 | 0.0288383 | 0.219513 | (8.51973)/(7.26538) | -2.44854 | -2.82156 | (1.07491)/(7.26538) | Cytoplasm | enzyme |
| ALPP | 1.17899 | 1.23213 | (96.0628)/(40.6153) | -1.84559 | -1.94045 | (11.1721)/(40.6153) | Plasma Membrane | phosphatase |
| ANPEP | 0 | 0.105801 | (1.19232)/(1.09968) | 4.63077 | 4.86923 | (34.123)/(1.09968) | Plasma Membrane | peptidase |
| AOX1 | 0 | 0.133012 | (0.152917)/(0.137765) | 1.33016 | 1.94272 | (0.572799)/(0.137765) | Cytoplasm | enzyme |
| APOE | 1.14086 | 1.55592 | (4.24542)/(1.42182) | -0.921718 | -1.61638 | (0.478918)/(1.42182) | Extracellular Space | transporter |
| ASNS | -1.05389 | -1.23052 | (3.98174)/(9.29025) | -0.863543 | -1.03235 | (4.79405)/(9.29025) | Cytoplasm | enzyme |
| AXL | -0.00472831 | -0.120505 | (6.92101)/(7.47362) | 1.40565 | 1.49929 | (22.3261)/(7.47362) | Plasma Membrane | kinase |
| C1orf116 | -1.07015 | -1.9397 | (0.0562474)/(0.223875) | 0 | -0.0127077 | (0.234771)/(0.223875) | Cytoplasm | other |
| C4BPA | -1.0358 | -2.08794 | (0.10348)/(0.463944) | -0.813789 | -1.70883 | (0.143971)/(0.463944) | Extracellular Space | other |
| CASP9 | 0.452835 | 0.545562 | (27.7131)/(18.8567) | -1.6161 | -1.76155 | (5.86863)/(18.8567) | Cytoplasm | peptidase |
| CCL20 | -0.773623 | -1.24138 | (1.63098)/(3.85903) | 1.22684 | 1.52915 | (11.8173)/(3.85903) | Extracellular Space | cytokine |
| CD274 | 0 | 0.399799 | (0.178413)/(0.132517) | 1.91475 | 2.59352 | (0.876092)/(0.132517) | Plasma Membrane | enzyme |
| CD55 | 0.43886 | 0.458296 | (497.816)/(359.911) | -1.08145 | -1.10731 | (176.452)/(359.911) | Plasma Membrane | other |
| CFI | -0.17322 | -0.478478 | (1.91541)/(2.65343) | -1.82812 | -2.28039 | (0.569718)/(2.65343) | Extracellular Space | peptidase |
| CLDN1 | 0 | 0.0939902 | (14.7027)/(13.6824) | 1.08852 | 1.17222 | (32.5781)/(13.6824) | Plasma Membrane | other |
| COL1A1 | 0.830889 | 0.911153 | (17.4102)/(9.19434) | -1.24733 | -1.36716 | (3.76281)/(9.19434) | Extracellular Space | other |
| CREB5 | 1.25115 | 1.46444 | (2.12476)/(0.763203) | 0 | 0.0424792 | (0.830565)/(0.763203) | Nucleus | transcription regulator |
| CRYAB | 1.80493 | 2.11059 | (18.2784)/(4.17984) | -1.51889 | -2.12118 | (0.993084)/(4.17984) | Nucleus | other |
| CSF1 | -0.0365573 | -0.193272 | (3.54572)/(4.02709) | 1.2287 | 1.35524 | (10.8902)/(4.02709) | Extracellular Space | cytokine |
| CXCL2 | 0 | -0.0489442 | (7.50852)/(7.71462) | 1.89061 | 2.0643 | (34.1416)/(7.71462) | Extracellular Space | cytokine |
| CXCR4 | 0.140859 | 0.182659 | (140.006)/(122.53) | -1.02823 | -1.08127 | (61.163)/(122.53) | Plasma Membrane | G-protein coupled receptor |
| DKK3 | 0.639996 | 0.861924 | (4.34712)/(2.37232) | -1.44908 | -1.81253 | (0.708766)/(2.37232) | Extracellular Space | cytokine |
| ECM1 | 1.33456 | 1.5288 | (10.499)/(3.60777) | 0.128247 | 0.351853 | (4.86707)/(3.60777) | Extracellular Space | transporter |
| EFEMP1 | -0.761097 | -0.978032 | (2.28804)/(4.4822) | 1.43942 | 1.58718 | (14.2403)/(4.4822) | Extracellular Space | enzyme |
| ENO2 | 0 | 0.00657771 | (31.1405)/(30.791) | -1.14177 | -1.24133 | (13.7524)/(30.791) | Cytoplasm | enzyme |
| ENO3 | 0.556839 | 0.585972 | (446.046)/(295.166) | -2.07592 | -2.12683 | (71.374)/(295.166) | Cytoplasm | enzyme |
| EPCAM | -0.202165 | -0.502714 | (2.46561)/(3.47362) | -1.09505 | -1.45909 | (1.32752)/(3.47362) | Plasma Membrane | other |
| EPHB2 | 0.10971 | 0.253713 | (4.96959)/(4.13928) | 1.4264 | 1.54964 | (12.8087)/(4.13928) | Plasma Membrane | kinase |
| EVI2B | -0.925415 | -2.95188 | (0.00969528)/(0.138388) | 1.96615 | 2.86039 | (1.13308)/(0.138388) | Plasma Membrane | other |
| F3 | 0.0684802 | 0.142588 | (36.7201)/(33.0407) | 1.56437 | 1.62653 | (107.78)/(33.0407) | Plasma Membrane | transmembrane receptor |
| FABP3 | 0.305249 | 0.37913 | (88.3177)/(67.4482) | -1.61108 | -1.72339 | (21.5628)/(67.4482) | Cytoplasm | transporter |
| FN1 | 0.447147 | 0.4839 | (45.643)/(32.4179) | 1.23096 | 1.2645 | (82.2678)/(32.4179) | Extracellular Space | enzyme |
| FOLR1 | 1.46167 | 1.50296 | (419.553)/(147.03) | -1.651 | -1.72247 | (47.0505)/(147.03) | Plasma Membrane | transporter |
| GAL3ST4 | 0.0853421 | 1.94294 | (0.12184)/(0.0250834) | 1.47161 | 3.13297 | (0.30195)/(0.0250834) | Cytoplasm | enzyme |
| GDF15 | 1.1628 | 1.27734 | (50.0799)/(20.5117) | -1.32227 | -1.50579 | (7.61907)/(20.5117) | Extracellular Space | growth factor |
| GDPD3 | -0.0602107 | -0.513756 | (1.6735)/(2.37904) | 1.2881 | 1.62129 | (7.77541)/(2.37904) | Cytoplasm | enzyme |
| GGT1 | 1.17637 | 1.32132 | (14.6738)/(5.8276) | -0.939281 | -1.15445 | (2.76139)/(5.8276) | Plasma Membrane | enzyme |
| GNG2 | 0 | -0.337167 | (0.0995475)/(0.125697) | 1.97861 | 2.65492 | (0.867359)/(0.125697) | Plasma Membrane | enzyme |
| GPRC5B | 0 | 0.155038 | (0.537091)/(0.477745) | 1.39511 | 1.84201 | (1.8303)/(0.477745) | Plasma Membrane | G-protein coupled receptor |
| GPX3 | 0 | 0.251407 | (0.263606)/(0.217076) | 1.69385 | 2.45762 | (1.31802)/(0.217076) | Extracellular Space | enzyme |
| HIST1H2BO | -1.10123 | -1.74398 | (1.32537)/(4.50212) | -1.2265 | -1.8894 | (1.25333)/(4.50212) | Nucleus | other |
| HIST1H4J | -1.19835 | -2.17006 | (0.706312)/(3.34069) | 0 | -0.525891 | (2.43783)/(3.34069) | Nucleus | other |
| HPGD | 0.913721 | 1.32403 | (1.6549)/(0.651685) | -1.36861 | -2.11927 | (0.153012)/(0.651685) | Cytoplasm | enzyme |
| HPX | 0 | 1.72995 | (0.119175)/(0.0261704) | 1.15947 | 2.96304 | (0.31089)/(0.0261704) | Extracellular Space | transporter |
| IDH1 | 0 | -0.00147328 | (118.411)/(117.74) | -1.02267 | -1.07278 | (59.119)/(117.74) | Cytoplasm | enzyme |
| IGFBP3 | -0.345384 | -0.402336 | (46.8077)/(61.4517) | -1.14315 | -1.21043 | (28.046)/(61.4517) | Extracellular Space | other |
| IL11 | 0 | 0.238807 | (2.21029)/(1.85849) | 1.55144 | 1.80693 | (6.89342)/(1.85849) | Extracellular Space | cytokine |
| IL8 | 0 | -0.0877794 | (4.10665)/(4.33464) | 2.09236 | 2.28389 | (22.3492)/(4.33464) | |  |
| INHBA | 0 | 0.144985 | (0.0889291)/(0.0781141) | 1.8329 | 3.13707 | (0.807319)/(0.0781141) | Extracellular Space | growth factor |
| ITGA11 | 1.17314 | 2.48775 | (0.235549)/(0.0381584) | 0 | 0.43253 | (0.0564108)/(0.0381584) | Plasma Membrane | other |
| ITGB8 | 0.0398219 | 0.291577 | (0.910138)/(0.737993) | 1.18388 | 1.40236 | (2.06498)/(0.737993) | Plasma Membrane | other |
| KRT1 | 0 | -0.607494 | (0.284094)/(0.433237) | -1.07582 | -2.12442 | (0.098815)/(0.433237) | Cytoplasm | other |
| KRT18 | -0.212766 | -0.227694 | (1237.87)/(1439.82) | -1.2492 | -1.26769 | (631.628)/(1439.82) | Cytoplasm | other |
| KRT75 | 0.964959 | 2.79408 | (0.200247)/(0.019988) | 3.72616 | 5.37113 | (1.30121)/(0.019988) | Cytoplasm | other |
| LAMC2 | 0.398386 | 1.03834 | (0.24687)/(0.117568) | 1.29305 | 1.87652 | (0.465546)/(0.117568) | Extracellular Space | other |
| LGR6 | 0.298424 | 0.506308 | (3.50708)/(2.45045) | -1.59312 | -1.92759 | (0.676462)/(2.45045) | Plasma Membrane | G-protein coupled receptor |
| LPL | 0 | -0.0171357 | (82.8814)/(83.3111) | -1.59609 | -1.65 | (28.0364)/(83.3111) | Cytoplasm | enzyme |
| MAMDC2 | 1.87548 | 3.17741 | (0.4758)/(0.0469591) | 0 | 0.999571 | (0.105991)/(0.0469591) | Extracellular Space | other |
| MGAT4A | -0.0317508 | -0.230754 | (1.25161)/(1.45905) | -1.3941 | -1.66069 | (0.485908)/(1.45905) | Cytoplasm | enzyme |
| MMP7 | -0.40211 | -0.732327 | (2.90391)/(4.80163) | -1.12404 | -1.51176 | (1.76759)/(4.80163) | Extracellular Space | peptidase |
| MPZL2 | 1.00033 | 1.16841 | (8.24437)/(3.63953) | -0.939732 | -1.18713 | (1.68508)/(3.63953) | Plasma Membrane | other |
| MUC13 | -0.177249 | -0.214779 | (104.976)/(121.014) | -1.1719 | -1.21789 | (54.9488)/(121.014) | Extracellular Space | other |
| NCCRP1 | 0.120459 | 0.306014 | (7.5192)/(6.03857) | -1.22484 | -1.48596 | (2.27097)/(6.03857) | Cytoplasm | other |
| NDRG1 | 0 | 0.0265886 | (82.9352)/(80.8763) | -1.7329 | -1.79178 | (24.6687)/(80.8763) | Nucleus | kinase |
| NT5E | 0 | -0.149404 | (4.20315)/(4.63064) | 1.05768 | 1.1895 | (11.1631)/(4.63064) | Plasma Membrane | phosphatase |
| PECAM1 | 1.06054 | 1.5085 | (0.938219)/(0.324306) | 0 | -0.617101 | (0.22209)/(0.324306) | Plasma Membrane | other |
| PRKCH | 0 | -0.535713 | (0.331011)/(0.478535) | 1.39606 | 1.80176 | (1.77882)/(0.478535) | Cytoplasm | kinase |
| PSCA | 0.547112 | 0.614638 | (99.6479)/(64.638) | -1.30741 | -1.40554 | (25.7632)/(64.638) | Plasma Membrane | other |
| PTX3 | -1.33217 | -1.60663 | (1.94619)/(5.90655) | 1.60001 | 1.76015 | (21.1619)/(5.90655) | Extracellular Space | other |
| PZP | 0 | 0.283061 | (1.28807)/(1.05035) | -1.53526 | -1.99164 | (0.275819)/(1.05035) | Extracellular Space | other |
| RAB3B | 0 | 0.0130194 | (0.0421659)/(0.0413369) | 2.3328 | 2.97368 | (0.355135)/(0.0413369) | Cytoplasm | enzyme |
| RPLP0P2 | 0.29695 | 1.2478 | (0.224972)/(0.0903714) | 1.54648 | 2.38673 | (0.526761)/(0.0903714) | Other | other |
| S100P | -0.163907 | -0.215639 | (311.446)/(359.243) | -1.59071 | -1.66119 | (119.95)/(359.243) | Cytoplasm | other |
| SEPP1 | 0.528556 | 0.768941 | (4.82016)/(2.80529) | -1.7658 | -2.19251 | (0.641311)/(2.80529) | Extracellular Space | other |
| SERPINE1 | -0.215639 | -0.367599 | (5.48462)/(7.03013) | 1.38929 | 1.50678 | (21.1157)/(7.03013) | Extracellular Space | other |
| SLC12A3 | -0.224506 | -0.273997 | (30.561)/(36.7065) | -1.14347 | -1.20324 | (16.8367)/(36.7065) | Plasma Membrane | transporter |
| SPINK1 | -1.10233 | -2.49816 | (0.350877)/(2.16707) | 0 | -0.466541 | (1.64759)/(2.16707) | Extracellular Space | other |
| TACSTD2 | -1.33523 | -2.95188 | (0.0373399)/(0.348488) | 0 | -0.327519 | (0.292224)/(0.348488) | Plasma Membrane | other |
| TAGLN | 0.262332 | 0.642074 | (2.55907)/(1.62314) | 1.33146 | 1.6686 | (5.48323)/(1.62314) | Cytoplasm | other |
| TCN1 | 2.46613 | 2.73888 | (14.6516)/(2.16845) | -1.10683 | -1.60071 | (0.74704)/(2.16845) | Cytoplasm | transporter |
| TGFB2 | 1.09859 | 1.22159 | (8.5048)/(3.62039) | 0.283563 | 0.419216 | (5.11523)/(3.62039) | Extracellular Space | growth factor |
| TGFBI | 0.807708 | 0.884784 | (39.4289)/(21.207) | 1.67962 | 1.75054 | (75.3929)/(21.207) | Extracellular Space | other |
| THBS1 | -0.60207 | -0.677748 | (11.0607)/(17.5767) | 1.08046 | 1.13779 | (40.8574)/(17.5767) | Extracellular Space | other |
| TNFAIP3 | 0.779042 | 0.91043 | (8.63673)/(4.5618) | 2.60426 | 2.71859 | (31.7454)/(4.5618) | Nucleus | enzyme |
| TNFRSF11B | 1.54377 | 1.80015 | (6.52981)/(1.85577) | -1.39613 | -1.86165 | (0.533395)/(1.85577) | Plasma Membrane | transmembrane receptor |
| TSPAN9 | 0.00641449 | 0.138177 | (6.36586)/(5.74495) | 1.24787 | 1.36055 | (15.5907)/(5.74495) | Plasma Membrane | other |
| VTN | 0.389085 | 0.447674 | (94.5421)/(68.8533) | -1.82188 | -1.91654 | (19.2563)/(68.8533) | Extracellular Space | other |

**Supplementary Table 1 The expression, location and type of 91 secreted genes out of 532 DEGs.** Group information: OE_group1 comprises SENP3 overexpressed L02 with and without FFA treatment; KD_group2 comprises SENP3 knocked-down L02 with and without FFA treatment. Gfold value with 0 means no statistical significance.
